# Correction: Ellsworth, M.; Ostrosky-Zeichner, L. Isavuconazole: Mechanism of Action, Clinical Efficacy, and Resistance. *J. Fungi* 2020, *6*, 324

**DOI:** 10.3390/jof11030226

**Published:** 2025-03-17

**Authors:** Misti Ellsworth, Luis Ostrosky-Zeichner

**Affiliations:** 1 Division of Pediatric Infectious Diseases, UT Health McGovern Medical School, Houston, TX 77030, USA; 2 Division of Infectious Diseases, UT Health McGovern Medical School, Houston, TX 77030, USA; Luis.Ostrosky-Zeichner@uth.tmc.edu

In the original article [1], in Section 6.3, “Treatment groups received caspofungin 750 mg IV on day 1” should be “70 mg” instead of “750 mg”. We made a mistake in the process of writing. The corrected paragraph is as follows:

“Treatment groups received caspofungin 70 mg IV on day 1 followed by 50 mg IV daily or 200 mg of isavuconazole IV q8h on days 1–2 followed by 200 mg once daily.”

The authors state that the scientific conclusions are unaffected. This correction was approved by the Academic Editor. The original publication has also been updated.
